# Biological Functions and Health Benefits of Flavonoids in Fruits and Vegetables: A Contemporary Review

**DOI:** 10.3390/foods14020155

**Published:** 2025-01-07

**Authors:** Xiaoyuan Zheng, Xuejiao Zhang, Fankui Zeng

**Affiliations:** 1 Research Center for Natural Medicine and Chemical Metrology, Lanzhou Institute of Chemical Physics, Chinese Academy of Sciences, Lanzhou 730000, China; zhengxiaoyuan@licp.cas.cn; 2 College of Food Science and Technology, Nanjing Agricultural University, Nanjing 210095, China; zhangxuejiao8@126.com

**Keywords:** flavonoids, function, human health, fruits and vegetables

## Abstract

Flavonoids, being prevalent in fruits and vegetables, are essential to the diverse stages of plant growth, development, and storage. Furthermore, flavonoids have been shown to exert substantial beneficial effects on human health, prompting heightened scientific interest in their potential advantages. This review elucidates the functions of flavonoids in fruits and vegetables, confirming their position as natural sources of these compounds, despite the differences in type and concentration among various species. This review elucidates the significance of flavonoids in the growth and development of fruits and vegetables, highlighting their roles in enhancing pigmentation and providing protection against both biotic and abiotic stresses. In relation to human health, flavonoids are recognized for their ability to combat aging, mitigate inflammation, safeguard the nervous system, and promote overall well-being. Additionally, this review proposes avenues for future research in the domain of flavonoids, underscoring the necessity for ongoing exploration of their potential applications and benefits.

## 1. Introduction

In recent years, as economic growth has improved living standards and raised health awareness, interest in the health benefits of natural compounds in fruits and vegetables has surged. Among these, flavonoids—a major class of secondary metabolites—have attracted significant attention for their potential as functional ingredients and natural food additives [1,2,3]. Structurally, flavonoids consist of a 15-carbon backbone (C6-C3-C6) comprising two benzene rings (A and B) connected by a three-carbon bridge. Key structural components include two-phenyl-chromones and two-phenyl-3-hydroxy-chromones, forming the basis of flavones and flavonols, respectively [4].

Flavonoids are abundant in many parts of plants, including leaves, flowers, roots, stems, and seeds [5]. Common subclasses present in fruits and vegetables encompass flavones, flavonols, isoflavones, anthocyanins, chalcones, dihydroflavonols, dihydroflavones, proanthocyanidins, and flavonoid carbonosides [6]. Among these, flavonols and flavones are particularly prevalent, with quercetin, kaempferol, isorhamnetin, apigenin, and luteolin as notable representatives [7]. The majority of flavonols exist as *O*-glycosides, with glucose as the predominant glycosidic sugar, followed by galactose, rhamnose, and, less commonly, xylose, arabinose, and glucuronic acid [8].

Flavones and flavonols also play crucial roles in plant biology, participating in growth, development, reproduction, UV protection, defense mechanisms, and flower pigmentation [1,8]. Beyond their ecological functions, flavonoids offer a range of health benefits, including anti-inflammatory, antioxidant, and anti-proliferative properties, which may help prevent diseases such as cancer and cardiovascular disorders [9,10].

While numerous studies have reviewed flavonoids’ chemical composition, nutritional value, pharmacological effects, and metabolic pathways, relatively few have examined their specific functions in fruits and vegetables [1,2]. Recent research has increasingly focused on the types and quantities of flavonoids present in these foods, as well as their role in plant growth and development. This review provides an updated overview of these developments, emphasizing flavonoids’ physiological roles in fruits and vegetables and their contributions to human health.

## 2. Principal Flavonoids in Fruits and Vegetables

More than 4000 distinct flavonoids have been identified in fruits and vegetables [11,12]. However, the types and concentrations of flavonoids vary widely among different produce [7]. Flavonoids are particularly abundant in green leafy, yellow, and red vegetables (e.g., onion, cabbage, cauliflower, and broccoli) and dark-colored fruits such as citrus fruits, berries, grapes, apples, and plums [13]. Leafy greens, cruciferous vegetables, and Allium species generally have high flavonoid levels, with leafy greens showing particularly strong antioxidant activity [9,14]. For instance, sweet potato leaves contain approximately 900 mg of quercetin per kg of fresh weight, while the total flavonoid content in potherb mustard and toona leaves is about 853 mg/kg and 805.7 mg/kg, respectively. Among cruciferous vegetables, pakchoi, kailan, Chinese cabbage, and cauliflower exhibit varying levels of flavonoids, from 1400 mg/kg to 180 mg/kg of fresh weight [9,15].

In the Allium genus, the flavonoid content is highest in leek (2140 mg/kg fresh weight), followed by garlic (960 mg/kg) and onion (280–540 mg/kg) [14,16]. High-flavonoid fruits include members of the Rosaceae, Rhamnaceae, Vitaceae, and Rutaceae families. Rosaceae fruits such as apples, nectarines, and plums contain flavonoid levels of 344.4, 111.2, and 127.2 mg/kg of fresh weight, respectively. Rhamnaceae fruits (dates) have a flavonoid concentration of 252.6 mg/kg, while in the Rutaceae family, mandarins and oranges contain 155.5 and 116.3 mg/kg, respectively. Green grapes from the Vitaceae family have a flavonoid content of 101 mg/kg [7].

Though many fruits are rich in flavonoids, their specific flavonoid profiles vary significantly (Table 1). Kaempferol, luteolin, and quercetin are among the most common flavonoids in fruits and vegetables, with kaempferol and quercetin especially prominent in vegetables and quercetin prevalent in fruits [7]. Kaempferol is found in broccoli, kale, garlic, cabbage, asparagus, gooseberries, strawberries, and several other vegetables and fruits [17,18]. Luteolin is high in garlic stalks, green lettuce, radishes, sweet potatoes, apples, pineapples, watermelons, and tomatoes [7]. Quercetin is abundant in sweet potatoes, onions, okra, kale, arugula, blueberries, peaches, blackberries, grapes, cherries, and plums [19].

## 3. Metabolic and Physiological Functions of Flavonoids in Fruits and Vegetables

Flavonoid production in plants begins early in bud development and continues through fruit ripening and maturity, playing essential roles in both plant biology and human health [20]. These compounds contribute to plant coloration, resistance to UV-B damage, cell wall structure, and disease defense and may also influence fertilization in some higher plants [21,22].

### 3.1. Role in Plant Development

The vibrant colors of petals in many fruit and vegetable plants are due to flavonoids, including yellow aurones, chalcones, and red or purple anthocyanins. Some petals also contain flavonols or flavanones, which, though colorless alone, alter flower color by forming complexes with anthocyanins and metal ions. This process, known as co-pigmentation, can produce striking blue hues. These floral pigments act as visual signals to attract pollinators, aiding in seed dispersal [23].

Flavonoids also play critical roles in plant reproduction. Found in pistils and pollen, flavonoids support seed formation by stimulating pollen production, germination, and pollen tube growth [22]. During anther maturation, flavonols accumulate in pollen exine pores and significantly promote germination and tube growth, as shown in tobacco pollen studies [24]. Experiments on CHS-silenced (RNA interference silencing of chalcone synthase) tomato plants, which have reduced flavonoid synthesis, resulted in parthenocarpic fruits—indicating flavonoids’ importance in normal pollen generation and tube growth [22].

Additionally, flavonoids such as quercetin, kaempferol, and bestatin regulate the transport of indole-3-acetic acid (IAA), an auxin critical for root development [25]. In tobacco plants, the silencing of flavonol synthase (FLS) led to reduced quercetin levels, increasing the polar transport of free IAA to the root and decreasing its concentration in the shoot apical region. This shift resulted in delayed flowering, smaller flowers, reduced pollen germination, and fewer seeds, underscoring quercetin’s role in auxin-mediated growth and reproduction [26].

### 3.2. Contribution to Color in Fruits and Vegetables

Flavonoids, particularly anthocyanins, contribute to the vibrant range of colors in fruits and vegetables beyond green, creating hues of orange, red, purple, and blue [27]. Among the 700 naturally occurring anthocyanins, six—pelargonidin, cyanidin, delphinidin, peonidin, petunidin, and malvidin—are found in nearly all fruits and vegetables [28].

The coloration effects of anthocyanin are influenced by several factors: pH, enzyme activity, cofactors, chelating agents, temperature, and light exposure [29]. Anthocyanin colors vary greatly with pH; at low pH, they appear red, shifting from the orange-red of pelargonidin anthocyanins to purple-red as hydroxylation increases, as seen with cyanidin and delphinidin. At higher pH, the colors shift from red to yellow and blue [30].

Light is a crucial factor for anthocyanin accumulation, as high light levels stimulate production. Fruits exposed to direct sunlight generally exhibit deeper anthocyanin colors compared to those in shaded areas [27]. Enzymes also play a role in anthocyanin degradation. For example, in red tart cherries, phenolase indirectly contributes to pigment loss by oxidizing phenolic substrates, which then degrade anthocyanins non-enzymatically [31].

Anthocyanins’ stability and color intensity can be affected by the presence of specific cofactors and metal ions. For instance, anthocyanins may chelate metals like aluminum, tin, copper, and iron, resulting in compounds that alter color intensity [32]. Temperature also impacts anthocyanins’ stability and synthesis; low temperatures can enhance anthocyanins’ production and color, while high temperatures generally inhibit synthesis and increase degradation rates, reducing anthocyanin concentrations [33].

### 3.3. Functions in Resisting Biotic Stresses

During growth, development, and postharvest storage, fruits and vegetables face numerous biological stressors. Flavonoids play key roles in protecting these plants from various biotic stresses.

#### 3.3.1. Deterrence of Animals and Weed Control

Flavonoids assist plants in coping with herbivore pressure by both repelling and attracting insects, some of which act as predators of herbivores. Certain flavonoids, such as hesperetin-7-*O*-rutinoside, quercetin-3-*O*-rutinoside, and naringenin, stimulate oviposition in specific insect species, like the swallowtail butterfly on citrus leaves [34]. Additionally, flavonoids like quercetin-3-*O*-rutinoside can both attract beneficial insects and repel herbivores [35].

Flavonoids also contribute to chemical defenses against herbivores. Compounds such as apigenin and 3,7-di-*O*-methylkaempferol, found in Cistus ladanifer exudates, inhibit Ca^2+^-ATPase in herbivore muscles, causing oral skeletal relaxation and avoidance [36]. Additionally, flavonoids secreted through plant roots into the surrounding soil can inhibit the germination of competing plant species by inducing oxidative stress and inhibiting auxin function, ultimately affecting seed germination and root development [37,38].

#### 3.3.2. Resistance to Pathogenic Fungi

Flavonoids exhibit strong antifungal activity and play crucial roles in defending plants against pathogens. Their antifungal mechanisms often leverage the antioxidant properties of flavonoids, helping neutralize reactive oxygen species (ROS) produced by both the plant and pathogen at infection sites [39]. Flavonoids can localize in cell walls of infected tissue to reinforce structural defenses and trigger hypersensitive responses that lead to programmed cell death around the infection site [40].

**Figure 1:**
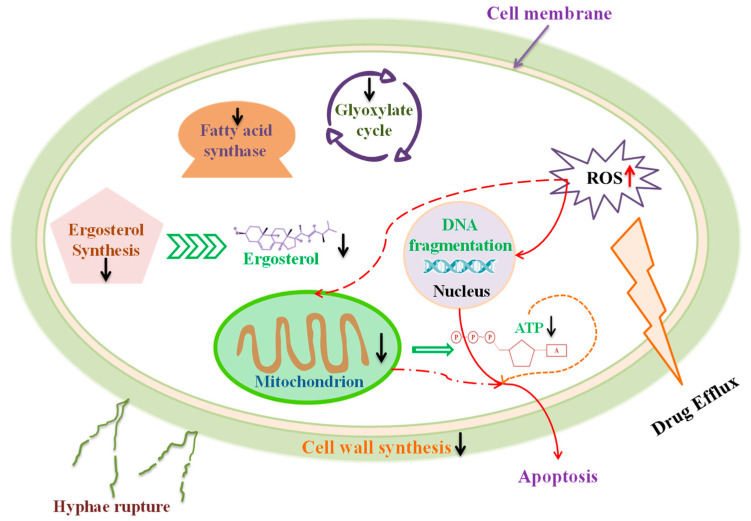
Mechanism of flavonoids inhibiting fungi. The up arrow indicates that the corresponding metabolism is activated, and the down arrow indicates that the corresponding metabolism is inhibited.

The antifungal mechanisms of flavonoids involve several aspects (Figure 1), such as the following:

(1) Inducing cellular apoptosis and mitochondrial dysfunction [39,41];
(2) Increasing intracellular ROS levels and inducing redox-related gene expression [41];
(3) Disrupting cell membranes, leading to cell shrinkage and the loss of intracellular contents [42];
(4) Causing DNA fragmentation and structural changes in fungal cells [41];
(5) Inhibiting ergosterol synthesis and protoplast regeneration [43];
(6) Repressing cell wall synthesis genes [43];
(7) Interfering with the glyoxylate cycle by inhibiting malate synthase gene expression [39].

#### 3.3.3. Resistance to Pathogenic Bacteria

The main cause of spoiling in fruits and vegetables that are ready to consume is pathogenic bacteria, which result in disagreeable sensory alterations. Flavonoids are naturally antimicrobial and studies have shown that they can work in concert with antibiotics to lessen the virulence of bacteria [44]. Flavonoids like quercetin and morin derivatives from *Psidium guajava* leaves exhibit bacteriostatic effects on foodborne pathogens, including *Listeria monocytogenes*, *Salmonella enterica*, and *Staphylococcus aureus* [45].

**Figure 2:**
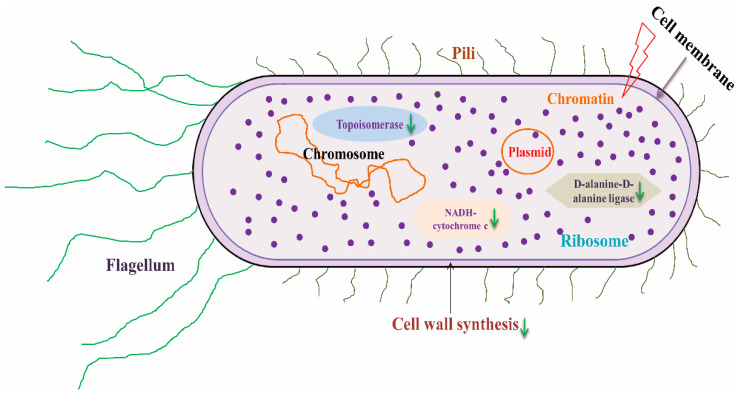
Mechanism of flavonoids inhibiting bacteria. The up arrow indicates that the corresponding metabolism is activated, and the down arrow indicates that the corresponding metabolism is inhibited.

The antibacterial mechanisms of flavonoids involve several aspects (Figure 2):

(1) Damaging the cytoplasmic membrane via pore formation or decreasing fluidity [46];
(2) Inhibiting nucleic acid synthesis by targeting topoisomerases [47];
(3) Disrupting energy metabolism via NADH-cytochrome c reductase inhibition [44];
(4) Blocking cell wall synthesis by inhibiting D-alanine-D-alanine ligase [48];
(5) Preventing cell membrane synthesis by inhibiting fatty acid biosynthesis enzymes such as FabG, FabI, FabZ, Rv0636, or KAS III [44].

All things considered, flavonoids greatly increase the biotic stress resistance of fruits and vegetables, improving their quality and longevity during development and storage.

### 3.4. Functions in Resisting Abiotic Stresses

Due to their immobility, plants must endure various environmental stresses, such as intense light, ultraviolet (UV) radiation, extreme temperatures, ozone, heavy metals, and drought [49]. To survive, plants have developed numerous defense mechanisms, including the production of secondary metabolites like flavonoids, which play key roles in mitigating the effects of these stresses [38].

Flavonoids, particularly those that absorb UV-B radiation, help plants resist the harmful effects of excessive light [4]. For example, UV-A radiation (wavelengths > 320 nm) can cause sunburn in apple skin, turning it brown, while UV-B radiation (280–320 nm) promotes reactive oxygen species (ROS) production. This ROS accumulation, also seen with general light stress, induces the biosynthesis of flavonoids, helping plants manage excess light exposure [50].

With the rise in extreme weather conditions, temperature fluctuations are becoming a critical stressor for crops. Research shows that tomatoes respond to temperature stress by increasing flavonoid production, enhancing their resilience [51]. Similarly, mango fruits high in anthocyanins and flavonoids exhibit greater resistance to chilling stress [52]. Salinity, another significant abiotic stressor, impedes the growth of many horticultural plants; however, increased flavonoid accumulation improves salt tolerance in strawberries [53].

Drought conditions also stimulate flavonoid and anthocyanin production in various plants. For example, water scarcity increases enzyme activities related to anthocyanin synthesis in strawberries, which helps the plant cope with drought [54]. Drought stress similarly induces flavonoid production in apple trees, providing enhanced resistance to water deficiency [55].

Physical damage is inevitable during the growth and handling of fruits and vegetables, and flavonoids accumulate at wound sites to prevent dehydration and manage oxidative stress. In potato tubers, for instance, wound-induced flavonoid accumulation helps scavenge ROS and limit water loss [56]. Flavonoid buildup also improves resistance to ozone, a potent oxidative pollutant, as seen in pepper fruits exposed to ozone stress [57]. Additionally, flavonoids help plants tolerate toxic metals like aluminum, allowing growth in metal-rich soils [58].

Abiotic stressors generally disrupt cellular redox homeostasis, leading to increased ROS production. This redox imbalance triggers flavonoid biosynthesis, providing plants with additional antioxidant protection [59]. Flavonoids contribute to the reduction of oxidative damage via multiple antioxidant mechanisms, such as the inhibition of oxidase enzymes, which aids in protecting cells from free radicals [60].

**Figure 3:**
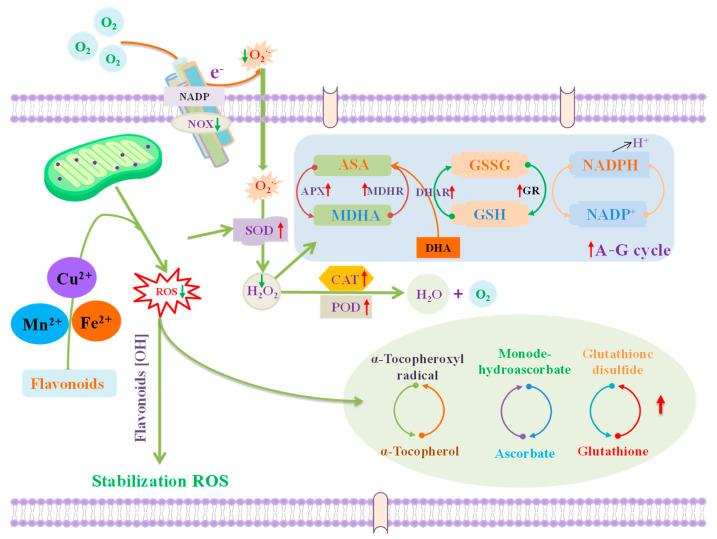
Antioxidant mechanisms of flavonoids in fruits and vegetables. The up arrow indicates that the corresponding metabolism is activated, and the down arrow indicates that the corresponding metabolism is inhibited.

Key antioxidant mechanisms (Figure 3) of flavonoids include the following:

(1) Direct ROS scavenging: Flavonoid molecules, especially those with an ortho-dihydroxy structure in the B ring, a 2,3-double bond conjugated with a 4-oxo group in the C ring, and hydroxyl groups at positions 3 and 5, are highly effective at scavenging radicals [61];
(2) Activation of antioxidant enzymes: Flavonoids can activate enzymes like NAD(P)H-quinone oxidoreductase, glutathione S-transferase, catalase, superoxide dismutase, and others, enhancing the plant’s antioxidant capacity [11];
(3) Metal chelation: Flavonoids chelate metal ions that catalyze oxidative reactions, thereby reducing ROS and protecting cells from metal-induced toxicity [62];
(4) Interaction with *α*-tocopherol radicals: Flavonoids help maintain α-tocopherol’s radical stability, slowing the oxidation of low-density lipoproteins and offering antioxidant protection [63];
(5) Inhibition of oxidases: By blocking enzymes like NADPH oxidase, cyclooxygenase, lipoxygenase, and others, flavonoids prevent excess ROS production, preserving cellular health [11].

## 4. Contribution to Human Health of Flavonoids in Fruits and Vegetables

### 4.1. Fruit and Vegetable Flavonoids in Diet to Promote Health

Fruits and vegetables play a vital role in our diets, supplying essential nutrients, vitamins, minerals, carbohydrates, and bioactive compounds like flavonoids, all of which are conducive to better health [7]. Numerous epidemiological studies suggest a link between high dietary flavonoid intake and a reduced risk of degenerative diseases, including cardiovascular disease, type 2 diabetes, atherosclerosis, and various cancers [64]. Flavonoids have broad health benefits, such as reducing chronic disease risk and enhancing immune function, along with antibacterial, antimalarial, anticancer, antiviral, antiangiogenic, and neuroprotective effects [1]. Recent research also highlights flavonoids’ anti-aging effects, suggesting they may promote longer, healthier lives [2]. Overall, incorporating flavonoid-rich foods and beverages into the diet supports general health and wellness.

### 4.2. Multiple Pharmacological Activities of Flavonoids

#### 4.2.1. Anticancer Activities

Flavonoids hold promise as both chemo-preventive and chemotherapeutic agents against diverse cancers. They exert anticancer effects through mechanisms such as cell cycle arrest, apoptosis, autophagy, and inhibiting cancer cell proliferation and metastasis [10]. Quercetin, a prevalent dietary flavonol, demonstrates anticancer properties by regulating pathways such as PI3K/AKT/mTOR, Wnt/β-catenin, and MAPK/ERK1/2, which trigger cell death and inhibit tumor growth. The PI3K/Akt/mTOR signaling pathway is a key pathway for regulating growth and metabolism, can integrate extracellular and intracellular signals, and is a classic pathway used in response to insulin signaling. The Wnt/β–catenin signaling pathway is a classical form of the Wnt signaling pathway which mainly involves the transcriptional activation of the *β*-Catenin. This pathway plays an important role in embryonic development and physiological processes of adult mammals, especially in stem cell function and disease development. The MAPK/ERK1/2 signaling pathway is one of the most important pathways in the eukaryotic signaling network. It is closely related to the occurrence of a variety of diseases, especially in the occurrence and proliferation of tumors [65,66]. In animal studies, quercetin has also shown the potential to reduce tumor proliferation and prolong survival in pancreatic cancer models [67]. Another flavonoid, luteolin, has been shown to inhibit cancer cell growth by promoting apoptosis, arresting the cell cycle, and reducing cell replication and invasiveness [68]. Apigenin, another potent flavonoid, targets cancer stem cells in multiple cancer types, including breast, prostate, and colorectal cancer, and can help overcome chemoresistance, improving the effectiveness of cancer treatments [69].

#### 4.2.2. Anti-Inflammatory Activities

Flavonoids are also significant for their anti-inflammatory effects. Inflammation, a natural immune reaction, can result in chronic diseases such as diabetes, cardiovascular disease, and cancer if left uncontrolled [70]. Flavonoids inhibit key enzymes in inflammatory pathways, such as phospholipase A2, cyclooxygenase, and lipoxygenase, which are involved in producing pro-inflammatory compounds like prostaglandins and thromboxane [71]. These bioactive compounds also counter ROS-mediated inflammation, which contributes to neurodegenerative diseases [72]. For instance, tomatoes, which are high in anti-inflammatory flavonoids, help raise total plasma antioxidant capacity, preventing chronic diseases brought on by inflammation [73]. Additionally, pumpkin flavonoids have been found to protect cells from inflammation caused by mycotoxins [74].

#### 4.2.3. Neuroprotective Activities

Because of their antioxidant qualities, flavonoids also help the neurological system by potentially postponing or avoiding neurodegenerative illnesses. These substances can upregulate genes that strengthen endogenous antioxidant defenses, trigger survival pathways, or directly scavenge free radicals [75,76]. Citrus flavonoids, particularly flavanols, flavonols, and isoflavones, exhibit neuroprotective effects, sometimes improving cognitive function and offering potential in developing “brain foods” [3]. Flavonoids like hesperetin in citrus and europinidin in onions show neuroprotective potential, specifically against diseases like Alzheimer’s [72,77]. Other flavonoids, such as luteolin from carrots and celery and rutin from asparagus, may offer protective benefits for Alzheimer’s disease [78,79].

#### 4.2.4. Anti-Aging Activities

Aging, a significant risk factor for many chronic diseases, is characterized by cellular decline often driven by oxidative stress [70]. Recent studies have shown that flavonoids can slow aging by promoting DNA repair, improving protein quality control, and reducing cellular damage [2]. For instance, quercetin combined with dasatinib has been found to reduce senescent cells and extend lifespan in mice [80]. The antioxidant properties of flavonoids, supported by structures with hydroxyl groups, help mitigate oxidative stress and may slow aging [81]. Naringenin and nobiletin, citrus flavonoids, reduce the ROS in senescent cells, contributing to anti-aging effects [82]. Additionally, specific flavonoids like diosmetin have been shown to activate pathways like NRF2/NQO1-HO-1, reducing oxidative damage in aging cells [83].

#### 4.2.5. Cardiovascular Disease Prevention

Epidemiological research conducted in the 1990s and 2000s has shown that flavonoids may reduce the risk of cardiovascular disease [84]. The flavonoids in foods might exert a strong antioxidant effect and reduce the morbidity and mortality of coronary heart disease. Studies have shown that daily dietary flavonoids can effectively reduce cardiovascular diseases caused by air pollution [85]. A previous study evaluating the intake of flavonoids in men aged 65 to 84 years illustrated that dietary flavonoid intake was significantly negatively correlated with death due to coronary heart disease and negatively weakly correlated with the incidence of myocardial infarction [86]. Because flavonoids lower phospholipase C and protein kinase C phosphorylation, limit platelet aggregation, and modify calcium mobilization, they exhibit strong antiplatelet and antithrombotic actions. They also reduce tissue factor expression and attenuate atherosclerosis development by decreasing oxidized LDL levels [87,88,89]. Research has also found that increasing intakes of healthy, flavonoid-rich foods may protect against atherosclerosis in the peripheral and carotid arteries [90].

## 5. Flavonoid Biodisponibility

In summary, flavonoids in fruits and vegetables offer a wide range of health benefits, with potential applications in cancer prevention, the prevention of cardiovascular disease, inflammation management, neuroprotection, and anti-aging, making them valuable in promoting human health. However, there is a problem of bioavailability when flavonoids function in vivo. Bioavailability usually refers to the part of digested flavonoids absorbed and metabolized through conventional ways [91]. According to reports, the small intestine can absorb 5–10% of the total intake of flavonoids, primarily those with monomeric and dimeric structures, frequently following deconjugation reactions like deglycosylation. The remainders make it to the colon, where the gut microbiota’s enzymatic activity further breaks them down into substances with various physiological significance [92]. The bioavailability of flavonoids is related to its structure. Flavonoid glycosides are generally of low bioavailability and only a few of them are absorbed into the blood; however, they still have definite efficacy, which may be due to their metabolites (secondary glycosides or agly-cones) entering the body to play important roles. As such, after oral administration, some flavonoids can be directly absorbed and others might be metabolized and transformed under the action of intestinal flora and liver metabolizing enzymes to generate active metabolites [93]. Harada et al., (2004) [94] compared the absorption of acylated and non-acylated anthocyanins and found that the acetylated anthocyanins were more easily absorbed. Previous research results showed that anthocyanins with more hydroxyl and less methoxy had a lower bioavailability and higher transport efficiency of glucoside than galactoside [95].

## 6. Conclusions and Perspectives

**Figure 4:**
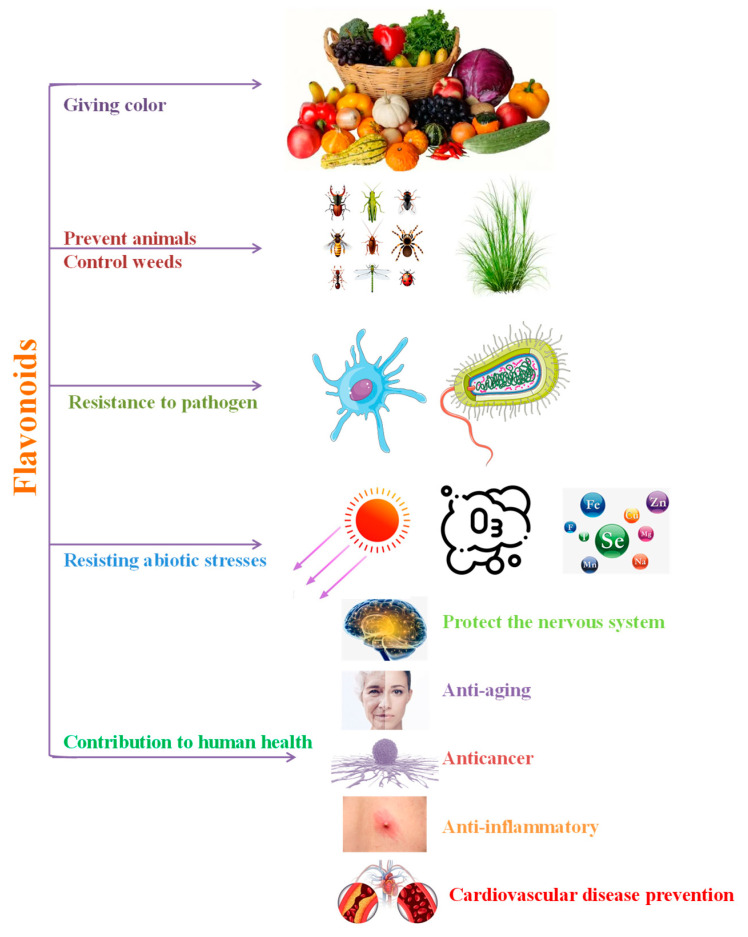
The function of flavonoids and its contribution to human health.

As the global consumption of fruits and vegetables increases, flavonoids in fruits and vegetables are becoming increasingly popular among researchers. This review details the functions of flavonoids in fruits and vegetables (Figure 4). First, fruits and vegetables need flavonoids to be fertile and reproduce sexually, influencing pollen development and pollen tube growth and giving flowers a beautiful color that attracts pollinators and helps seed dispersal. In addition, flavonoids control endogenous free IAA transport and have an impact on how plants develop their roots. However, there are few studies on flavonoids in fruit and vegetable growth and development and many mechanisms need to be further investigated, which may provide new ideas for the breeding of new fruit and vegetable varieties in the future.

Flavonoids give more color to fruits and vegetables, thus increasing consumers’ enjoyment of fruits and vegetables. Additionally, flavonoids in fruits and vegetables support their ability to withstand a variety of biotic and abiotic challenges. During their growth and development, fruits and vegetables are inevitably exposed to a variety of abiotic stresses, which mainly lead to an imbalance of reactive oxygen species. The homeostasis of reactive oxygen species in the cells of fruits and vegetables during growth and development is maintained by flavonoids, which have potent antioxidant activities and can protect against abiotic stressors. Even though a lot of research has been done on the ability of flavonoids to withstand biotic and abiotic challenges, there is still work to be done on the best ways to make use of flavonoids in fruits and vegetables to help them play a bigger part in this respect.

As dietary flavonoids, the role of flavonoids in fruits and vegetables in promoting human health should not be overlooked. Most of the flavonoids in fruits and vegetables have strong biological activity. Flavonoids have been shown to have good anticancer activity, especially quercetin, luteolin, and apigenin, and they provide good resistance to a variety of cancers, including breast, prostate, colorectal, and pancreatic cancers. Inflammation may cause diseases such as diabetes, asthma, and cardiovascular disease and, again, flavonoids have good anti-inflammatory activity. Flavonoids may also mitigate damage to the human nervous system, thereby preventing or postponing the occurrence of neurodegenerative diseases. In particular, citrus flavonoids are able to exert neuroprotective effects at different doses, so citrus fruits are expected to be used to develop neuroprotective and brain foods based on general foods. Furthermore, anti-aging has been at the vanguard of scientific research and flavonoids have been demonstrated to possess anti-aging characteristics. Simultaneously, flavonoids in fruits and vegetables are anticipated to be developed into novel functional foods and the modification of dietary flavonoids via metabolic engineering is a highly promising endeavor.

## Figures and Tables

**Table 1:** Category structure and main sources of flavonoids in fruits and vegetables.

Classes of Flavonoids	Subclasses of Flavonoids	Chemical Structure	Sources Rich
Flavanols	catechin epocatechin gallo-catechin epigallocatechin epigallocatechin gallate	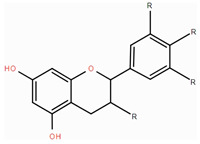	apples, cherries, plums, apricots, and berries and grape skins
Flavanones	naringenin eriodictyol hesperetin pinocembrin prunin	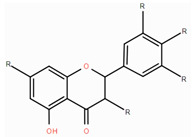	citrus, orange, and lemon
Anthocyanidins	cyanidin delphinidin pelargonidin malidin petunidin	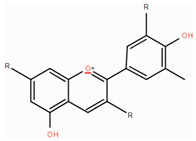	blueberries, red cabbage, tomato, purple sweet potato, and eggplant
Flavonols	kaempferol quercetin myricetin rutin robinin	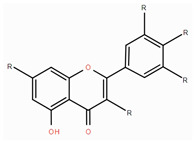	broccoli, onions, asparagus, and apples
Flavones	apigenin baicalein luteolin diosmetin tangeretin	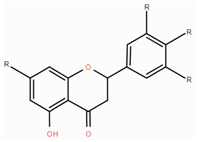	celery, red pepper, and oranges
Isoflavones	biochanin a formononetin daidzein genistein glycitein	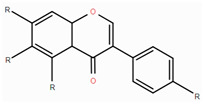	small amounts in many fruits and vegetables

## Data Availability

No new data were created or analyzed in this study. Data sharing is not applicable to this article.
